# Benzodiazepine prescription for patients in treatment for drug use disorders: a nationwide cohort study in Denmark, 2000–2010

**DOI:** 10.1186/s12888-016-0881-y

**Published:** 2016-05-27

**Authors:** Christian Tjagvad, Thomas Clausen, Marte Handal, Svetlana Skurtveit

**Affiliations:** Norwegian Centre for Addiction Research [SERAF], University of Oslo, Kirkeveien 166, Bygg 45, 0407 Oslo, Norway; Department of Pharmacoepidemiology, Norwegian Institute of Public Health, Oslo, Norway

**Keywords:** Benzodiazepines, Drug use disorders, Opioids, Cannabis, Central stimulating drugs, Long-term prescription, Addictive medication, Drug treatment, Cohort study, Denmark

## Abstract

**Background:**

Benzodiazepines are frequently prescribed to patients with drug use disorders. However, it has previously been difficult to distinguish whether this frequent prescribing was due to underlying psychiatric disorders or inappropriate prescribing. In a nationwide cohort study, we investigated the prescribing of benzodiazepines to patients with drug use disorders in connection with treatment admission.

**Methods:**

Benzodiazepine prescriptions to patients (N = 33203) aged 18 to 67 years admitting for outpatient treatment for drug use disorders in Denmark, 2000 to 2010, were studied by using linked data from nationwide health registries. Factors associated with increasing amounts of benzodiazepine use within the first year after admission were assessed by multinomial logistic regression. Proportions of very long-term benzodiazepine prescription were calculated.

**Results:**

During the first year after admission to treatment, 26.2 % of patients were prescribed benzodiazepines. Of these, 35.5 % were prescribed benzodiazepines at dose levels that might indicate inappropriate use (>365 Defined Daily Dose per year), and 34.6 % were prescribed more than one type of benzodiazepines. Diazepam was the most commonly prescribed type. Among patients with opioid use, 43.2 % were prescribed benzodiazepines which were three times higher than for patients with cannabis (12.2 %) or central stimulating drugs (13.8 %) as their primary drug use. Admitting to treatment for a drug use disorder did not increase the specialized psychiatric treatment coverage of this patient group, disregarding use of prescribed benzodiazepines. 29.5 % were new users of prescribed benzodiazepines, and of these, 27.5 % continued into very long-term use (≥4 years after admission) during the study period.

**Conclusions:**

Benzodiazepines were commonly prescribed to patients admitting to treatment for drug use disorders, and included prescription of multiple and non-optimal types, high doses, and very long-term prescriptions. These findings point towards inappropriate prescribing of benzodiazepines in many cases more than treatment for psychiatric disorders.

## Background

Drug use disorders (DUDs) are common medical conditions and associated with significant morbidity and mortality [1]. Treatment of DUD reduces illicit drug use, morbidity, mortality, and crime [2–5]. In recent years, outpatient treatment of DUD has largely replaced previous traditions of inpatient treatment [6].

Benzodiazepines (BZDs) have long been used for conditions including various psychiatric disorders, insomnia, acute alcohol withdrawal, and epilepsy [7]. However, the use of BZDs has also been associated with a risk for dependence, abuse, and overdose death [8]. Patients with DUD use BZDs more frequently than the general population, including both legally prescribed and illicitly acquired BZDs [9]. Further, this patient group has both a higher prevalence of psychiatric disorders and an increased risk of developing a BZD abuse when using the medication [10–13]. Therefore, it has previously been difficult to distinguish whether the excess use of BZDs, particularly legally prescribed BZDs, among patients with DUD has been due to underlying psychiatric disorders or abuse; two conditions which require entirely different treatment regimes.

The prescribing of BZDs is mainly approved for short- to intermediate-term treatment in low doses. Benzodiazepines are not indicated for long-term use, except in the case of severe generalized anxiety disorder [14, 15]. In particular, long-term use among patients with DUD is of increasing concern, as it can result in cognitive impairment, difficulty in continuing treatment, tolerance, and dependence and abuse of BZDs [16–18]. Furthermore, the risk for adverse events, such as dependence and abuse, differs among the different types of BZDs. Certain types are preferred among drug using populations, and are often used in combination with other drugs [17, 19, 20]. BZDs used in combination with opioids may be particularly counterproductive in this patient group due to high risk of abuse [21]. Therefore, long-term prescriptions, high doses, less than optimal types of BZDs, and co-prescribing of opioids are all likely indicators of inappropriate prescribing of BZDs. On the other hand, initiation of psychiatric treatment for BZD users after admission to DUD treatment would be a likely indicator of an underlying psychiatric disorder. Investigation of such indicators among patients with DUD may help clarify whether the excess use of BZDs among this patient group is due to an underlying psychiatric disorders or inappropriate prescribing.

There are an estimated 33000 drug users in Denmark (latest numbers from 2009) with relatively stable treatment coverage of around 40 % during the last decades [6]. The outpatient treatment for patients with DUD is comprised of multiple service components, including acute detoxification, medication-assisted treatment, case management, and behavioral therapy. According to Danish guidelines, BZDs should not be prescribed to patients with DUD as a general rule [22]. Once admitted into treatment, guidelines state that DUD treatment should address the patient’s use of BZDs, and that BZDs should not be prescribed without coordinating within the overall treatment plan [22]. From 2000 to 2010, the prevalence of BZD use in the general Danish population decreased considerably, with an average one-year prevalence of 5-6 % during the entire period [23–25].

The Danish national registries contain information on patients seeking treatment for DUD, psychiatric disorders, and dispensed prescription drugs. In this nationwide study, we aimed to investigate the prescribing of BZDs in a population admitted for treatment of DUD. For patients with and without prior use of BZDs, we examined how their primary drug use and if psychiatric treatment prior to admission influenced BZD prescribing one year after admission. Finally, we assessed both the proportion of the different types of BZDs prescribed and the very long-term BZD prescription rates after admission into treatment.

## Methods

### Study population

**Fig. 1:**
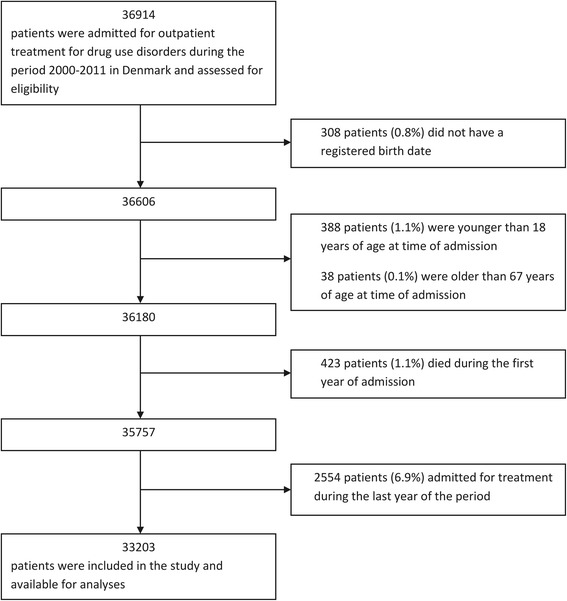
Flow chart indicating the procedure for generating the study population

This study included 33203 patients aged between 18 and 67 years old who were consecutively admitted for public outpatient treatment for DUD between January 1, 2000 and December 31, 2010 (Fig. 1).

### Data sources

This study was based on data from the Danish Substance Abuse Treatment Register (DSATR) [26], the Danish National Prescription Database (DNPR) [27], and the Danish Psychiatric Central Research Register (PCRR) [28]. Data was obtained for the period of January 1, 1999 to December 31, 2011. All data sources were linked by use of the personal identification number, a unique identifier assigned to all Danish residents since 1968 [29].

The DSATR was used to identify patients in treatment for different DUDs. The register was established in 1996 and contains information on all patients receiving treatment in publically funded outpatient drug treatment facilities in Denmark. All treatment in these facilities is provided free of charge to the patient, as Denmark provides access to universal health care (including DUD treatment) for all residents. There are few privately funded drug treatment facilities, except for treatment of alcohol use disorders. Therefore practically all patients in treatment for DUDs are included in this study. The date of admission into treatment was registered. Patients are registered with one self-reported primary drug of abuse used upon admission into treatment, where patients are asked about “primary drug” in relation to treatment needs. Besides illicit non-prescription drugs, prescription drugs such as methadone, buprenorphine, and BZDs could also be reported as their “primary drug” of abuse.

The DNPR contains information on all individual prescription drugs dispensed through pharmacies in Denmark since 1994 to patients outside of institutions such as hospitals and drug treatment facilities. Information about all prescriptions from ambulatory care, whether publicly reimbursed or not, is stored in the DNPR. Drugs are classified according to the Anatomical Therapeutic Chemical (ATC) classification [30]. The study used prescription data about BZDs, opioid analgesics, opioid maintenance medication (methadone, buprenorphine), and Z-hypnotics from the DNPR, which cover all patient prescriptions in Denmark. The data collected for this study included; patient unique identifying number (encrypted), gender, the date the medication was dispensed, and medication information [brand name, ATC-code, and defined daily dose (DDD)].

Benzodiazepines were defined by the ATC-code N05BA, N05CD, and N03AE01 in the ATC-classification system. Opioid analgesics were defined by the ATC-code N02A. Methadone and buprenorphine prescribed for opioid maintenance treatment were defined by the ATC-code N07BC02 and N07BC01/N07BC51, respectively. Z-hypnotics were defined by the ATC-code N05CF. Methadone and buprenorphine are used for both opioid maintenance treatment and pain treatment in Denmark. The present study included prescriptions of methadone and buprenorphine when these medications were prescribed for either indication. In this paper the terms ‘prescribed drug’ and ‘used drug’ are used interchangeably to describe dispensed drugs at pharmacies. For each prescription, the numbers of DDD dispensed were recorded. A DDD is defined as the assumed average maintenance dose per day for a medication used for its main indication in adults (Table 1) [30, 31].

**Table 1:** ATC code and Defined Daily Dose (DDD) of prescribed benzodiazepines^a^

	ATC code	1 DDD (oral administration)	584 DDD (oral administration)
Alprazolam	N05BA12	1 mg	584 mg
Bromazepam	N05BA08	10 mg	5840 mg
Chlordiazepoxide	N05BA02	30 mg	17520 mg
Clobazam	N05BA09	20 mg	11680 mg
Clonazepam	N03AE01	8 mg	4672 mg
Diazepam	N05BA01	10 mg	5840 mg
Estazolam	N05CD04	3 mg	1752 mg
Flunitrazepam	N05CD03	1 mg	584 mg
Flurazepam	N05CD01	30 mg	17520 mg
Lorazepam	N05BA06	2.5 mg	1460 mg
Midazolam	N05CD08	15 mg	8760 mg
Nitrazepam	N05CD02	5 mg	2920 mg
Oxazepam	N05BA04	50 mg	29200 mg
Triazolam	N05CD05	0.25 mg	146 mg

Abbreviations: ATC, Anatomical Therapeutic Chemical

^a^Intake of 1 DDD daily of benzodiazepines in a 1 year period results in a total intake of 365 DDD

Information about psychiatric diagnosis was obtained from the PCRR. The PCRR contains data on all psychiatric hospitalizations in Denmark since 1970, psychiatric ambulatory visits and emergency department contacts since 1995. Discharge/contact diagnoses from the PCRR are coded according to ICD-10. A patient was only identified as having received psychiatric treatment if a diagnosis with the ICD-10 code F01-F99 was given during treatment. For certain analyses, these ICD-10 codes were further grouped into seven categories.

### Analysis strategy

Study entry was set as the date of first admission into treatment for DUD during the study entry period, 2000–2010. We followed patients during the first year (365 days) after admission with respect to BZD prescription. Patients in the upper quartile received a yearly BZD dose of ≥584 DDD. The patients were categorized into two groups by amount prescribed during the first year after admission: (i) yearly dose <584 DDD (moderate-high dose) and (ii) yearly dose ≥584 DDD (very high dose). First, we compared characteristics at the time of admission between groups with and without a prescription for BZDs in moderate-high and very high doses. Further, for these groups we investigated the proportion of patients with a prescription for other potentially addictive drugs and psychiatric diagnoses. We assessed the proportion (%) with 95 % confidence intervals (CI) of different categories of psychiatric diagnoses that were given to patients with and without a prescription for BZDs in moderate-high and very high doses during the first year after admission.

Second, a prescription of BZDs during the year prior to admission into treatment may likely influence the prescription pattern after admission. Therefore we performed the following analyses in two different strata. In the first strata, we included patients with at least one prescription of BZDs in the year prior to admission (previous users). In the second strata, we included patients who had not received a BZD prescription in the year prior to admission, but who had at least one prescription of BZDs in the year after admission (new users). Variables potentially associated with prescription of BZDs in moderate-high and very high doses were estimated by multinomial logistic regression for both strata.

Third, we assessed the proportion of different types of BZDs in the moderate-high and the very high dose categories for both previous and new users. Finally, we investigated very long-term prescription of BZDs for previous and new users in 2000–2007 (N = 5374 and N = 2122, respectively). For this analysis, patients were consecutively excluded if they had died during the year of assessment (total; n = 510 and n = 120, respectively). A very long-term prescription of BZDs was defined as having had at least one prescription of BZDs in at least four consecutive years after the admission date, and included long-term sporadic use. We calculated the median and interquartile range (IQR 25 to 75 %) of DDDs of BZDs in the fourth year for each group.

### Statistical analysis

All analyses were conducted using SPSS version 19.0 for Windows. To test for differences between groups, Chi-square analysis and t-test were used. Unadjusted and adjusted relative risk ratio (RRR and aRRR) with 95 % CI was estimated by multinomial regression analysis. In multinomial regression analysis, gender, age group at study entry date, primary drug use, and psychiatric diagnosis within 1 year prior to study entry were included in the model as independent variables. The level of significance was set to P < 0.05.

### Ethics

The Danish Data Protection Agency has approved all procedures in relation to data collection from all used databases, and also storage of the data. The National Committee on Health Research Ethics in Denmark was informed about the study and determined that the study did not need to be reported to the Committee. All linkages were performed within Statistics Denmark, a governmental institution that collects and maintains electronic records for a broad spectrum of statistical and scientific purposes.

## Results

A total of 33203 patients between the ages of 18 to 67 years were admitted for outpatient treatment for DUDs during January 1, 2000 until December 31, 2010. The mean age was 31 years (SD ± 11) at first admission, and 8177 (24.6 %) were female. During the first year after admission 8705 patients (26.2 %) were prescribed BZDs. The yearly mean dose prescribed was 438.1 DDD during the first year after admission, and the median was 175.0 DDD (interquartile range, 40.0 to 584.5). Of the patients with a BZD prescription, 3086 (35.5 %) were prescribed BZDs in a yearly dose higher than 365 DDD during the year after admission.

Table 2 compares characteristics in the groups with and without a BZD prescription in the different dosage categories at the time of admission. Age at admission was highest in the group with the highest amounts of prescribed BZDs (mean 41.0 years). The proportion of females was higher in groups with a BZD prescription compared to the group without a BZD prescription. Patients with opioids reported as their primary drug used were overrepresented in the group with the highest amounts of prescribed BZDs (Table 2). The proportion of patients with opioids as their primary drug used was 53.6 % in this group compared to 40.4 % in the group with the lowest amounts of BZDs, and 20.4 % in the group without a BZD prescription. Conversely, the patients with cannabis or central stimulating drugs reported as their primary drug used were overrepresented in the group without a prescription for BZDs. During the year prior to admission the proportion of patients receiving psychiatric treatment was lowest for the group without BZD prescription (16.9 %) and highest in the group with the lowest amounts of BZDs (27.3 %). Among patients without a BZD prescription during the year after admission into treatment, 10.1 % were prescribed BZDs in the year prior to admission. However, in the groups with a prescription of BZDs after admission in lower and higher amounts, 63.0 % and 93.1 % respectively, were prescribed BZDs in the year prior to admission (Table 2).

**Table 2:** Characteristics of patients at the time of admission into treatment for DUDs, by prescription and dose category of benzodiazepines

	No prescription of benzodiazepines 1 year after admission		Prescription of benzodiazepines 1 year after admission in moderate-high doses <584 DDD^a^		Prescription of benzodiazepines 1 year after admission in very high doses ≥584 DDD^a^		*p* value
	N	(%)	N	(%)	N	(%)	
Number of individuals	24498		6528		2177		
Age in years at admission date, mean (SD)		29.2 ± 9.7		35.3 ± 10.7		41.0 ± 9.5	<0.001
Gender							<0.001
Male	18884	77.1	4630	70.9	1512	69.5	
Female	5614	22.9	1898	29.1	665	30.5	
Primary drug use at admission							<0.001
Cannabis	9212	37.6	1193	18.3	84	3.9	
Opioids	5011	20.4	2639	40.4	1166	53.6	
Methadone or buprenorphine	1236	5.0	955	14.6	594	27.3	
Heroin	3454	14.1	1458	22.3	463	21.3	
Other opioids	321	1.3	226	3.5	109	5.0	
Benzodiazepines	213	0.9	207	3.2	83	3.8	
Central stimulating drugs^b^	3648	14.9	534	8.2	50	2.3	
Other illicit drugs/unknown	6414	26.2	1955	29.9	794	36.5	
Psychiatric treatment received in 1 year prior to admission^c^	4148	16.9	1782	27.3	430	19.8	<0.001
Prescription of benzodiazepines 1 year prior to admission	2464	10.1	4113	63.0	2026	93.1	<0.001

Abbreviations: DUDs, drug use disorders; DDD, Defined Daily Dose

^a^Intake of 1 DDD daily of benzodiazepines in a 1 year period results in a total intake of 365 DDD

^b^Amphetamine, cocaine, and MDMA

^c^Information obtained from the Danish Psychiatric Central Research Register

During the year after admission, the proportion of patients with a prescription of potentially addictive drugs (opioid analgesics, opioid maintenance medication, and Z-hypnotics) was highest in the group with the highest amounts of BZDs and lowest in the group without a BZD prescription (Table 3).

**Table 3:** Prescription of other addictive drugs and psychiatric treatment for patients in the first year after admission into treatment for DUDs

	No prescription of benzodiazepines 1 year after admission		Prescription of benzodiazepines 1 year after admission in moderate-high doses <584 DDD^a^		Prescription of benzodiazepines 1 year after admission in very high doses ≥584 DDD^a^		*p* value
	N	(%)	N	(%)	N	(%)	
Number of individuals	24498		6528		2177		
Prescription of other drugs 1 year after admission							
Opioid analgesics	1677	6.9	1323	20.3	668	30.7	<0.001
Opioid maintenance medication (methadone, buprenorphine)	2903	11.9	2271	34.8	1238	56.9	<0.001
Z-hypnotics	1489	6.1	1444	22.1	334	15.3	<0.001
Psychiatric treatment received in 1 year after admission^b^	3791	15.5	1825	28.0	431	19.8	<0.001

Abbreviations: DUDs, drug use disorders; DDD, Defined Daily Dose

^a^Intake of 1 DDD daily of benzodiazepines in a 1 year period results in a total intake of 365 DDD

^b^Information obtained from the Danish Psychiatric Central Research Register

During the year after admission, only the psychiatric diagnosis “mental and behavioral disorders due to psychoactive substance use” was more common in the groups with prescription of BZDs compared to the group without prescription of BZDs (Table 4).

**Table 4:** Psychiatric diagnoses (ICD-10 code: F00-F99) given to patients in psychiatric treatment in the first year after admission into treatment for DUDs^a^

	No prescription of benzodiazepines 1 year after admission		Prescription of benzodiazepines 1 year after admission in moderate-high doses <584 DDD^b^		Prescription of benzodiazepines 1 year after admission in very high doses ≥584 DDD^b^	
	N = 3791		N = 1825		N = 431	
	N	% (95 % CI)	N	% (95 % CI)	N	% (95 % CI)
Mental and behavioural disorders due to psychoactive substance use (ICD-10 code: F10–F19)	1821	48.0 (46.4-49.6)	1127	61.8 (59.5-64.0)	259	60.1 (55.3-64.7)
Schizophrenia and related disorders (ICD-10 code: F20–F29)	362	9.5 (8.6-10.5)	226	12.4 (10.9-14.0)	47	10.9 (8.2-14.3)
Mood disorders (ICD-10 code: F30–F39)	473	12.5 (11.5-13.6)	217	11.9 (10.5-13.5)	31	7.2 (5.0-10.2)
Neurotic, stress-related, and somatoform disorders (ICD-10 code: F40–F48)	629	16.6 (15.4-17.8)	296	16.2 (14.6-18.0)	63	14.6 (11.5-18.4)
Disorders of adult personality and behaviour (ICD-10 code: F60–F69)	390	10.3 (9.4-11.3)	194	10.6 (9.3-12.2)	32	7.4 (5.2-10.4)
Behavioural and emotional disorders with onset in childhood (ICD-10 code: F90–F98)	266	7.0 (6.2-7.9)	53	2.9 (2.2-3.8)	4	0.9 (0.3-2.5)
Other mental disorders (ICD-10 code: F00–F09, F50–F59, F70–F89, F99)^c^	253	6.7 (5.9-7.5)	104	5.7 (4.7-6.9)	26	6.0 (4.1-8.8)
Total^d^	4194		2253		462	

Abbreviations: DUDs, drug use disorders; DDD, Defined Daily Dose

^a^Information obtained from the Danish Psychiatric Central Research Register

^b^Intake of 1 DDD daily of benzodiazepines in a 1 year period results in a total intake of 365 DDD

^c^F00–F09: Organic, including symptomatic, mental disorders; F50–F59: Behavioural syndromes associated with physiological disturbances and physical factors; F70–79: Mental retardation; F80–89: Disorders of psychological development; F99: Unspecified mental disorder

^d^It is possible for one patient to be given more than one type of psychiatric diagnosis

From 2000 to 2010, the proportion of previous and new users with a BZD prescription during the first year after admission reduced continuously from 71.4 % and 18.1 % to 62.2 % and 5.9 %, respectively (data not shown).

With respect to factors associated with having a BZD prescription during the year after admission, the analyses were performed stratified according to the BZD prescription prior to admission (Tables 5 and 6). In both strata, no association after adjustment was observed between female gender and the prescription of very high amounts of BZDs. In the strata with new users of BZDs, females were more likely than males to be prescribed BZDs in moderate-high amounts (aRRR = 1.2, 95 % CI 1.1-1.3). Differences between strata in the association between age and BZD prescription in moderate-high and very high amounts were observed. In the strata with previous users of BZDs (Table 5), the RRR increased with increasing age in both dose categories (oldest age group: aRRR = 3.3, 2.7-3.9; aRRR = 7.8, 6.2-9.9). However in the strata with new users of BZDs, the RRR was increased only in older age groups among patients with prescription of BZDs in moderate-high amounts (aRRR = 3.3, 2.7-3.9).

**Table 5:** Relative risk ratio (RRR) from multinomial logistic regression of having a continued prescription of benzodiazepines according to different factors

	Prescription of benzodiazepines 1 year after admission in moderate-high doses <584 DDD^a^			Prescription of benzodiazepines 1 year after admission in very high doses ≥584 DDD^a^		
	Unadjusted RRR	Adjusted^b^ RRR (95 % CI)	*p* value	Unadjusted RRR	Adjusted^b^ RRR (95 % CI)	*p* value
Female	1.1	1.0 (0.9-1.2)	0.310	1.2	1.0 (0.9-1.2)	0.639
Age group at admission date						
18 – 27 years	1.0 (referent)	1.0 (referent)		1.0 (referent)	1.0 (referent)	
28 – 37 years	1.8	1.7 (1.4-1.9)	<0.001	3.8	2.8 (2.3-3.5)	<0.001
38 – 47 years	2.4	2.1 (1.8-2.4)	<0.001	7.6	4.9 (4.0-6.1)	<0.001
48 – 67 years	3.7	3.3 (2.7-3.9)	<0.001	13.3	7.8 (6.2-9.9)	<0.001
Primary drug use at admission						
Cannabis	1.0 (referent)	1.0 (referent)		1.0 (referent)	1.0 (referent)	
Methadone or buprenorphine	2.4	1.8 (1.5-2.2)	<0.001	15.2	8.3 (6.2-11.1)	<0.001
Heroin	1.5	1.3 (1.1-1.6)	0.001	5.1	3.6 (2.7-4.8)	<0.001
Other opioids	2.0	1.5 (1.1-2.1)	0.005	9.1	5.2 (3.6-7.7)	<0.001
Benzodiazepines	2.5	2.1 (1.5-2.8)	<0.001	9.4	6.9 (4.5-10.6)	<0.001
Central stimulating drugs^c^	0.9	0.9 (0.7-1.1)	0.431	1.1	1.1 (0.7-1.7)	0.633
Other illicit drugs/unknown	1.7	1.4 (1.2-1.6)	<0.001	7.6	4.6 (3.5-6.0)	<0.001
Psychiatric treatment received in 1 year prior to admission^d^	1.0	1.2 (1.0-1.3)	0.006	0.6	0.6 (0.5-0.7)	<0.001

Group of previous users without prescription of benzodiazepines, n = 2464; group of previous users with prescription of benzodiazepines in moderate-high doses, n = 4113; group of previous users with prescription of benzodiazepines in very high doses, n = 2026.

Abbreviations: DDD, Defined Daily Dose; CI, confidence interval.

^a^Intake of 1 DDD daily of benzodiazepines in a 1 year period results in a total intake of 365 DDD.

^b^Adjusted for gender, age group at admission date, primary drug use at admission, and psychiatric treatment received in 1 year prior to admission.

^c^Amphetamine, cocaine, and MDMA.

^d^Information obtained from the Danish Psychiatric Central Research Register.

**Table 6:** Relative risk ratio (RRR) from multinomial logistic regression of having a new prescription of benzodiazepines according to different factors

	Prescription of benzodiazepines 1 year after admission in moderate-high doses <584 DDD^a^			Prescription of benzodiazepines 1 year after admission in very high doses ≥584 DDD^a^		
	Unadjusted RRR	Adjusted^b^ RRR (95 % CI)	*p* value	Unadjusted RRR	Adjusted^b^ RRR (95 % CI)	*p* value
Female	1.2	1.2 (1.1-1.3)	<0.001	0.8	0.8 (0.5-1.2)	0.818
Age group at admission date						
18 – 27 years	1.0 (referent)	1.0 (referent)		1.0 (referent)	1.0 (referent)	
28 – 37 years	1.7	1.4 (1.2-1.5)	<0.001	4.1	1.9 (1.3-2.9)	0.003
38 – 47 years	1.9	1.3 (1.2-1.5)	<0.001	3.8	1.2 (0.7-2.0)	0.488
48 – 67 years	1.9	1.3 (1.1-1.5)	0.006	4.0	1.1 (0.6-2.2)	0.710
Primary drug use at admission						
Cannabis	1.0 (referent)	1.0 (referent)		1.0 (referent)	1.0 (referent)	
Methadone or buprenorphine	4.2	3.8 (3.2-4.4)	<0.001	52.9	45.7 (20.9-99.9)	<0.001
Heroin	2.7	2.5 (2.2-2.9)	<0.001	20.3	17.1 (8.0-36.5)	<0.001
Other opioids	2.8	2.4 (1.7-3.3)	<0.001	22.5	20.3 (6.5-63.7)	<0.001
Benzodiazepines	4.2	3.8 (2.7-5.4)	<0.001	14.2	15.1 (3.2-71.7)	0.001
Central stimulating drugs^c^	1.1	1.0 (0.9-1.2)	0.572	1.9	1.9 (0.7-5.6)	0.224
Other illicit drugs/unknown	1.5	1.4 (1.3-1.7)	<0.001	5.3	5.2 (2.3-11.4)	<0.001
Psychiatric treatment received in 1 year prior to admission^d^	1.4	1.5 (1.4-1.7)	<0.001	0.5	0.7 (0.4-1.3)	0.320

Group without prescription of benzodiazepines, n = 22034; group of new users with prescription of benzodiazepines in moderate-high doses, n = 2415; group of new users with prescription of benzodiazepines in very high doses, n = 151

Abbreviations: DDD, Defined Daily Dose; CI, confidence interval

^a^Intake of 1 DDD daily of benzodiazepines in a 1 year period results in a total intake of 365 DDD

^b^Adjusted for gender, age group at admission date, primary drug use at admission, and psychiatric treatment received in 1 year prior to admission

^c^Amphetamine, cocaine, and MDMA

^d^Information obtained from the Danish Psychiatric Central Research Register

Patients with opioids (methadone and buprenorphine; heroin) reported as their primary drug used were more likely to be prescribed BZDs in moderate-high and particularly very high doses, as compared to cannabis as their primary drug used in the strata of new BZD users (very high dose: aRRR (methadone and buprenorphine) = 45.7, 95 % CI 20.9-99.9; aRRR (heroin) = 17.1, 8.0-36.5) (Table 6). A similar but less strong association was observed in the strata of previous BZD users. Receiving psychiatric treatment prior to admission reduced the risk of being prescribed BZDs in very high doses in both strata (however, not significant for new users).

For both previous and new BZD users, the proportion of patients with prescription of more than one type of BZD was lowest for patients with prescription of BZDs in the lowest doses (29.2 %; 18.6 %) and highest for patients with prescription of BZDs in the highest doses (62.0 %; 66.9 %) (Table 7). Diazepam was the most commonly prescribed type of BZD to previous users (34.8 %), whilst oxazepam was the most common type prescribed to new users (28.6 %). However, among both of these groups with prescription of BZDs in only very high doses, nitrazepam was the most commonly prescribed type (53.7 %; 72.8 %).

**Table 7:** Types of benzodiazepines prescribed to previous and new users in the first year after admission for treatment for DUDs

	Prescription of benzodiazepines to previous users in the first year after admission				Prescription of benzodiazepines to new users in the first year after admission			
	Moderate-high doses <584 DDD^a^ N = 4113		Very high doses ≥584 DDD^a^ N = 2026		Moderate-high doses <584 DDD^a^ N = 2415		Very high doses ≥584 DDD^a^ N = 151	
Benzodiazepine type	N	%	N	%	N	%	N	%
Alprazolam	398	9.7	287	14.2	200	8.2	32	21.2
Bromazepam	303	7.4	450	22.2	101	4.2	30	19.9
Chlordiazepoxide	692	16.8	189	9.3	624	25.8	20	13.2
Clonazapam	739	18.0	252	12.4	305	12.6	14	9.3
Diazepam	1224	29.8	914	45.1	484	20.0	50	33.1
Flunitrazepam	82	2.0	201	9.9	46	1.9	18	11.9
Nitrazepam	756	18.4	1087	53.7	393	16.3	110	72.8
Oxazepam	1284	31.2	329	16.2	692	28.7	43	28.5
Other	186	4.5	188	9.3	104	4.3	13	8.6
Total^b^	5664	137.7	3897	192.3	2949	122.1	330	218.5
Multiple	1202	29.2	1257	62.0	449	18.6	101	66.9

Abbreviations: DUDs, drug use disorders; DDD, Defined Daily Dose

^a^Intake of 1 DDD daily of benzodiazepines in a 1 year period results in a total intake of 365 DDD

^b^It is possible for one patient to be prescribed more than one type of benzodiazepine

**Fig. 2:**
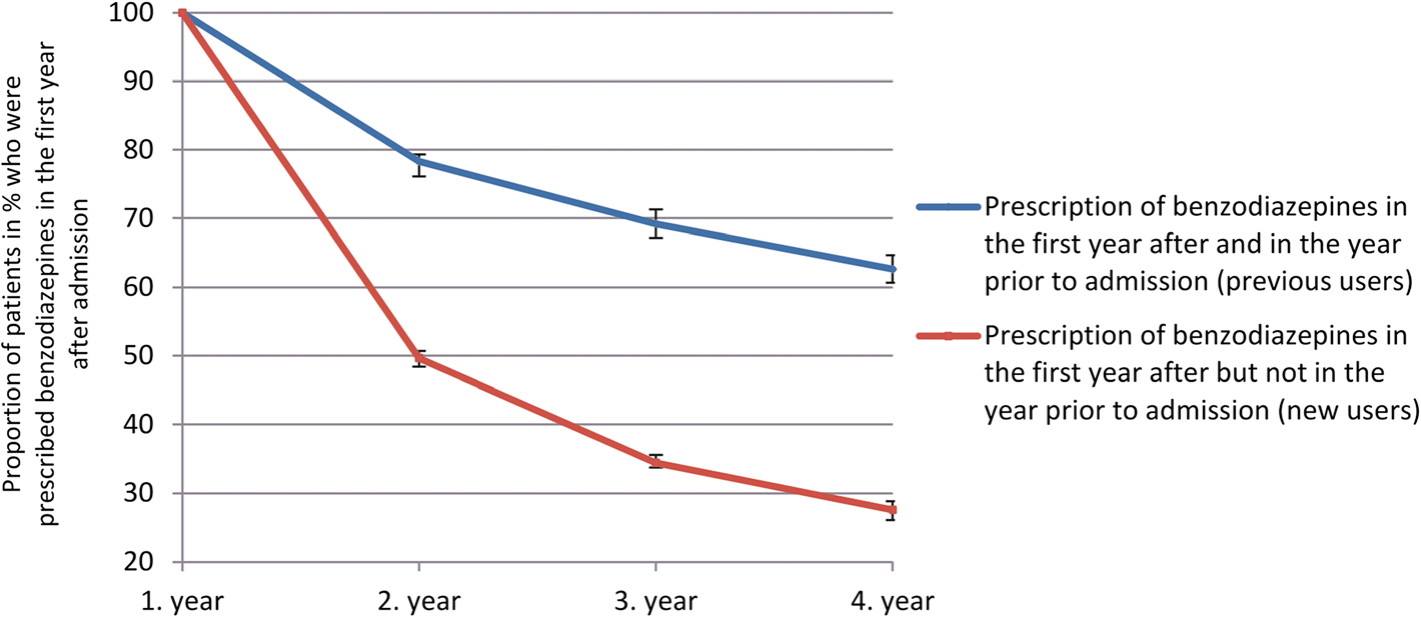
Proportion of patients (%) with very long-term prescription of benzodiazepines after admission for treatment for drug use disorders

A high proportion of patients with previous prescription of BZDs continued into very long-term use of prescribed BZDs after admission (4 years after admission: 62.6 %) (Fig. 2). Even in the group of patients without previous prescription of BZDs, 27.5 % continued into very long-term use. During the first year of prescription, the median and interquartile range of DDD was 343.9 (IQR 83.3 to 797.0); 50.0 (18.0 to 166.7) for previous and new users of BZDs, respectively. During the fourth year of prescription, the median and interquartile range of DDD was 405.0 (IQR 122.0 to 937.5); 118.8 (30.0 to 416.8) for previous and new users of BZDs, respectively.

## Discussion

In this large nationwide study involving 33203 Danish patients admitted during an 11-year period to outpatient treatment for all types of DUDs, one out of four received at least one BZD prescription during the first year after admission. Of these, 29 % were new users of prescribed BZDs. Among patients with an opioid as their primary drug used, 43 % were prescribed BZDs during the first year after admission, which was three times higher than for patients with cannabis or central stimulating drugs as their primary drug used. Diazepam was the most commonly prescribed type of BZD. Collectively, the findings point toward a prescribing practice of BZDs that overall does not follow clinical guidelines, and indicate an inappropriate prescription of benzodiazepines in many cases more than treatment for psychiatric disorders.

Our findings are consistent with the results of other studies showing that having a DUD increases the risk of BZD use [32]. Particularly, a high prevalence of BZD use among patients in treatment for opioid use disorder corresponds to the findings reported elsewhere [9, 33]. Use of BZDs in high doses was observed among patients with an OMT medication, methadone or buprenorphine, as primary drug used. This finding is consistent with the results of studies showing that users of OMT medication were prescribed higher doses of BZDs as compared to users of heroin [14].

Compared to the general population in the same age group and same time period in Denmark (one year prevalence 5-6 %) [23], the prevalence of BZD use was five times higher among patients with all types of DUD, and eight times higher among patients with opioids as their primary drug used. Although a decreasing trend in use of BZDs was observed in our study, the prevalence of BZD use in 2010 was approximately four times higher among patients with all types of DUD as compared to the general population. This excess BZD use among patients with DUD may therefore be of continued relevance after the study period.

In contrast to the general population, where female gender predicts BZD use, our study indicates that overall there was no association between gender and use of BZDs [34]. However, in consistency with the general population, use of BZDs was overall more common in older age groups compared with younger [25, 35].

Many of the patients admitted for DUD treatment require treatment for their harmful use of non-prescribed BZDs [36, 37]. The cessation of such non-prescribed BZDs is complex and often requires medical support, which can include the prescribing and tapering of BZDs [38]. In our study, more than one quarter of all users of BZDs were new users. Of these, one out of four continued with a very long-term prescription. If a prescription of BZDs is initiated, it should normally not exceed four weeks, or a tapering down period should be agreed upon with the patient prior to treatment to avoid a long-term prescription [22]. For high-risk drug users and particularly opioid users, a short tapering down period with frequent medication pickup should be considered [39].

The amount of BZDs prescribed in this study was measured by use of DDD (Defined Daily Dose). The therapeutic amount of BZD for its main indication is defined as 1 DDD per day, equivalent to 365 DDD per year [30]. In our study, one-third were prescribed above 365 DDD per year, which indicates inappropriate use of BZD. Prescription of BZDs in high doses should in general be avoided, particularly for patients with DUD due to the high risk for abuse. Involvement of specialized psychiatric care might reduce the prescribing of BZDs in high doses by offering a psychiatric diagnostics that could inform an alternative and more effective therapeutic offer [40]. Our results support this as receiving psychiatric treatment prior to admission seemed to reduce the risk of being prescribed BZDs in very high doses.

Each type of BZD possess different effects, and their appeal to drug users differ accordingly [17]. In our study, diazepam was the most commonly prescribed type of BZD. This prescribing practice may be less than optimal, as diazepam has been reported as attractive among drug users given its fast onset and superior euphoriant effect compared to other types [17, 41, 42]. In addition to diazepam, other types of BZDs were prescribed, and one-third were prescribed more than one type of BZD. Use of more than one BZD at a time may indicate inappropriate use of BZDs [43]. This is supported by our findings as the majority of patients receiving BZDs in very high doses were prescribed more than one type of BZD.

The strengths of this study were that it covers all patients admitted to treatment for all types of DUD in a nationwide study. The unique personal identity number provides high quality linkages between the population-based registries on DUD treatment, prescriptions, and psychiatric treatment in Denmark. There were no missing outcome variables of prescribed medications due to complete registry linkage.

The limitations of this study were that medications dispensed at hospitals and other institutions, sometimes without individual prescriptions, are not registered at an individual level and hence are not included in this study. Benzodiazepines prescribed from drug treatment facilities to alleviate alcohol withdrawal symptoms are overall not included in the study, as BZDs prescribed with such an indication most often are dispensed from the treatment facility and not registered in the Danish National Prescription Database. Further, dispensed medications from pharmacies are not necessarily consumed by the recipients, and this study cannot account for persons who may have given away or sold their prescribed medications. If the type of BZD prescribed to one patient consecutively changed during a year it may have been registered as being prescription of multiple types of BZD. However, a high proportion of patients with prescription of multiple types of BZDs were receiving very high doses which could indicate use of more than one type at a time. Only OMT (opioid maintenance treatment) medication dispensed from a pharmacy was included in this study, as OMT medication dispensed from a drug treatment facility is not registered in the Danish National Prescription Database. Psychiatric diagnoses exclusively from general practitioners, drug treatment facilities, or somatic hospitals are not in registered the Danish Psychiatric Central Research Register and therefore not included in this study. Further, patients may have used a combination of drugs where choice of one primary drug use can be difficult and lead to misclassification.

## Conclusions

The studied population in this nationwide sample included all patients with a DUD. In that view, regardless of the primary drug used, patients had too high prescription rates of BZDs, and this particularly applied to patients with an opioid use disorder. Our findings therefore have relevance for all physicians involved in DUD treatment.

Given that BZDs only have a very narrow indication as psychiatric treatment for this patient group, the current treatment as presented in this study seems less than optimal. This is underlined by the finding that admitting into treatment for DUDs with prescribed BZDs did not seem to increase specialized psychiatric care involvement. No difference regarding the prevalence of anxiety disorders have been reported among patients with different types of DUDs [12]. Still, the proportion of BZD users was three times higher among patients treated for opioid use when compared to patients treated for use of cannabis or central stimulating drugs. Opioids combined with BZDs are known to induce a greater level of euphoria, as opposed to cannabis and central stimulating drugs. Further, inappropriate prescription patterns of BZD were identified in this study with prescription of multiple and non-optimal types, high doses, very long-term prescription, and co-prescription of other potentially addictive drugs.

Overall, BZDs were prescribed to DUD patients in a fashion that in many cases indicated inappropriate prescribing to patients more than treatment for psychiatric disorders. This finding might reflect a liberal prescribing practice among physicians at drug treatment facilities in Denmark, which has previously been described regarding other addictive medications [44, 45]. Further, it emphasizes the potential risk of harm when BZD prescribing practices do not follow clinical guidelines. Our results reinforce the need for health systems to promote the use of prescription drug monitoring programs to identify inappropriate BZD prescribing patterns among patients, and help physicians link such patients to more appropriate care. Patients with patterns of inappropriate BZD use deserve medical support while tapering off their BZD dependence. DUD patients with comorbid psychiatric disorders in need of long-term BZD prescription are likely a minority, and co-management of specialized psychiatric care should be considered part of an appropriate BZD treatment for this patient group. For the remainder of the patients, physicians would likely benefit their patients if BZDs are avoided rather than prescribed; however to hold the restrictive role is more of a challenge than to be a liberal prescriber.

## Abbreviations

DUD, drug use disorders; BZDs, benzodiazepines; DSATR, Danish Substance Abuse Treatment Register; DNPR, Danish National Prescription Database; PCRR, Danish Psychiatric Central Research Register; ATC, Anatomical Therapeutic Chemical; CI, confidence intervals; DDD, defined daily dose; IQR, interquartile range; RRR, relative risk ratio; aRRR, adjusted relative risk ratio; OMT, opioid maintenance treatment.
